# Migration and health study: a socio-ecological analysis of sexual health among migrants in Manitoba, Canada

**DOI:** 10.1186/s12889-023-17379-9

**Published:** 2023-12-06

**Authors:** Rusty Souleymanov, Bolaji Akinyele-Akanbi, Chinyere Njeze, Patricia Ukoli, Paula Migliardi, John Kim, Michael Payne, Laurie Ringaert, Gayle Restall, Linda Larcombe, Nathan Lachowsky, Mohammad Nuruzzaman Khan, Robert Lorway, Fritz Pino

**Affiliations:** 1 Faculty of Social Work, University of Manitoba, 173 Dafoe Road West, Tier Building, office 500 C, Winnipeg, Winnipeg, MB R3T 2N2 Canada; 2 Department of Community Health Sciences, Rady Faculty of Health Sciences, University of Manitoba, Winnipeg, MB Canada; 3 Winnipeg Regional Health Authority, Winnipeg, MB Canada; 4 National Laboratory for HIV Reference Services, Public Health Agency of Canada, Winnipeg, MB Canada; 5 Nine Circles Community Health Centre, Winnipeg, MB Canada; 6 Manitoba HIV-STBBI Collective Impact Network, Winnipeg, MB Canada; 7 Department of Occupational Therapy, Rady Faculty of Health Sciences, University of Manitoba, Winnipeg, MB Canada; 8 Department of Internal Medicine, Rady Faculty of Health Sciences, University of Manitoba, Winnipeg, MB Canada; 9 Community Based Research Centre, Vancouver, BC Canada; 10 School of Public Health and Social Policy, University of Victoria, Victoria, BC Canada; 11 Faculty of Social Work, University of Regina, Regina, SK Canada

**Keywords:** Sexual health, Immigrants and refugees, HIV, Manitoba, Canada, Socio-ecological analysis

## Abstract

**Background:**

To develop effective public health policies, programs, and services tailored to the unique sexual health needs of migrant populations, it is essential to understand the myriad socio-ecological factors that influence their sexual health. This qualitative community-based participatory study aimed to explore factors influencing migrants’ sexual health at different socio-ecological levels in a Canadian setting.

**Methods:**

Participants (n = 34) from African, Caribbean, Black; Latin American; South Asian; Middle Eastern, as well as East and Southeast Asian communities were recruited across Manitoba using printed flyers, community organizations, and social media. Individual interviews, conducted in English, French, Mandarin, Cantonese, Tagalog, Arabic, Swahili, and Tigrinya languages, explored questions relating to sexual health and experiences with service providers. Data were analyzed using reflexive thematic analysis and socio-ecological systems theory.

**Results:**

The study uncovered a range of individual, interpersonal, institutional, and socio-structural factors that affect the sexual health of migrants in Manitoba. Individual factors such as sexual health knowledge and testing practices, interpersonal factors like the type of sexual partnerships, institutional factors such as sexual health information needs, language, and service access barriers, and structural-level factors like gender norms and HIV stigma exerted a significant influence on the sexual health practices of study respondents. Sexual health awareness was influenced by various factors including length of time in Canada and involvement in community-based services. Study respondents identified issues related to access to HIV testing and sexual health information, as well as language barriers, racism in healthcare, and HIV stigma. Gender and social norms played a significant role in discouraging communication about sex and safer sex practices.

**Conclusions:**

The study highlights the complex interplay of factors that influence the sexual health of migrants, and the need for targeted sexual health awareness campaigns and provision of sexual health information in languages spoken by migrants. Public health interventions focused on improving the sexual health outcomes for migrants should consider the socio-ecological elements identified in this study. These findings can inform public health campaigns to increase access to services and address sexual health inequities among migrant communities in Canada.

## Background

Migrant health, a critical 21st-century issue [1], necessitates a comprehensive examination of the intricate links between migration and sexual health. Gaining this understanding is crucial for guiding public health policies aligned with sustainable development goals [2, 3]. In order to develop effective public health policies, programs, and services tailored to the unique sexual health needs of migrant populations, it is imperative to deepen our understanding of the myriad of factors that influence their sexual health across various socio-ecological levels.

The socio-ecological theory model [4–18] is highly relevant to understanding the sexual health of migrant communities in Manitoba, emphasizing the interconnectedness of factors at individual (micro), interpersonal (meso), institutional (exo), and societal (macro) levels. The theory underscores the dynamic relationship between individual behaviors, social networks, community environments, and socio-structural factors [6, 7]. At the individual level, migrants face health risks influenced by age, education, and pre-migration health [6, 7]. Interpersonal influences include social networks and relationships [8, 9]. Institutional and community factors, such as healthcare accessibility and social environments also impact migrant health [10]. O’Campo and Dunn [11] emphasize the importance of understanding migrants’ social context, illustrating how environments influence access to care, and migrants’ needs shape their experiences. The framework extends to the structural level, considering socio-political contexts and their impact on migrants’ health, encompassing social inequities, determinants of health, structural violence, discrimination, and marginalization, which contribute to health disparities [8, 9, 12–15].

Researchers like Meunier and Agyemang [16], Gray et al. [17], and Organista et al. [18] increasingly apply a socio-ecological framework to study migrant sexual health. Meunier and Agyemang conducted a systematic review emphasizing the need for multi-level interventions that consider the socio-ecological complexities of migrants’ lives. Gray et al. explored factors contributing to sexual health disparities among migrant Asian women, highlighting the necessity for culturally sensitive interventions. Organista et al. critiqued HIV prevention efforts among Mexican migrants, also emphasizing the importance of multi-level interventions addressing the socio-ecological context of migrants’ lives and work. Research examining the socio-ecological elements of sexual health among migrants in Canada remains limited, particularly in the realm of qualitative research. The significance of this study, conducted in Manitoba, lies in its contribution to the body of research that is largely devoid of Canadian-focused investigations.

### Migrant health disparities: a Canadian focus

In Canada, migrants fall into four admission classes: economic immigrants, family-sponsored immigrants, temporary workers, and refugees [19]. Manitoba, particularly Winnipeg, attracts migrants seeking economic opportunities, experiencing rapid population growth [19]. In Canada, immigrants (economic or family class) generally have better health outcomes than refugees, but subgroup differences exist based on gender, country of origin, and residency time [20]. Migrant communities, vulnerable to sexual health inequities like human immunodeficiency virus (HIV) and sexually transmitted and blood borne infections (STBBI), face barriers such as service access, socio-economic challenges, language barriers, and intersecting oppressions [21–27]. Canadian research indicates refugees are twice as likely to have HIV compared to immigrants [22]. New migrants experience distinct sexual health inequities compared to long-term residents [24]. Studies outside Canada reveal structural drivers influencing HIV/STBBI transmission among migrants from different regions [28–31]. Scholars also explore factors such as condom availability, partner numbers [22], migration paths, integration, access to resources [24, 28, 30], social norms, HIV stigma [26], racism [23, 31], and settlement-related stress [24, 29] to understand migrants’ sexual health outcomes.

Recent articles [32–36] highlight healthcare challenges for immigrants and refugees in high-income countries. Brandenberger et al. [32] identified three key challenges: contextual (legal and financial barriers, lack of documentation, limited social support), communication (language barriers, inadequate interpretation, low health literacy), and cultural (beliefs, discrimination by providers). Hamed et al. [33] focused on healthcare racism, revealing interpersonal, institutional, and structural racism, leading to disparities. Davidson et al. [34] highlighted barriers to sexual health care for immigrant and refugee women, including knowledge gaps, financial constraints, cultural norms, language barriers, and fear of discrimination. Baidoobonso et al. [35] explored HIV risk among Black communities in Canada, uncovering disparities based on gender, age, sexual orientation, and migration status. Causevic et al. [36] examined sexual risk behaviors among young migrants, particularly those with limited access to sexual health information and services, revealing engagement in risky behaviors, such as unprotected sex, multiple partners, and transactional sex. Despite this knowledge, there is a significant research gap in understanding the sexual health of these communities, especially in Canadian Prairie contexts. This qualitative community-based participatory study aimed to explore factors influencing migrants’ sexual health at different socio-ecological levels in Manitoba, Canada.

## Methods

### Study design

This study used qualitative design and a community-based research approach. The socio-ecological model guided the formulation of interview questions which aimed to explore the multi-level factors influencing the sexual health of migrants, encompassing individual, interpersonal, community, and societal dimensions. During the analysis phase, the socio-ecological model served as a conceptual lens, facilitating the interpretation of findings in the context of interconnected levels and systems influencing sexual health outcomes among migrant populations.

The study was derived from, and included, an active Community Advisory Committee (CAC) that guided this research. CAC consisted of ten individuals with lived experience of migration, who guided the implementation, recruitment, and knowledge mobilization on this study. Involving the CAC in the study design and implementation was crucial for several reasons. First, it ensured that the study was culturally appropriate and sensitive to the needs of the migrant community in Manitoba. The CAC members were able to provide insights into the unique challenges and barriers faced by migrants in accessing healthcare and information about sexual health. Second, involving the CAC helped to build trust and establish rapport with potential participants. The CAC members were able to communicate the goals and objectives of the study to their respective communities, and to encourage participation in the research. Third, the CAC members played a key role in the analysis and interpretation of the data, which helped to ensure that the findings accurately reflected the perspectives and experiences of the participants.

### Participant recruitment, sampling, and eligibility

From August 2021 to January 2022 participants (n = 34) from African and Caribbean; Latin American; South Asian; as well as East and Southeast Asian communities were recruited from across Manitoba’s cities/town (Winnipeg, Brandon, Steinbach, and Thompson) using printed flyers, community agencies, social media, and word of mouth. Eligibility included: (1) identify as a migrant (including immigrants and refugees), (2) report any sex with another person in the previous 12 months, (3) be 18 years of age or older, (4) live in Manitoba. The study was approved by the University of Manitoba Research Ethics Board (HS23993 –P2020:092). Informed written consent was obtained from all participants. All data were kept confidential.

### Data collection and measures

Individual interviews were conducted at community-based organizations in English by three research assistants and the principal investigator. Interpretive services were available in Arabic, French, Mandarin, Cantonese, Tagalog, Swahili, and Tigrinya. Semi-structured interviews lasted approximately 90 min in duration. The interviewers comprising individuals with diverse experiences and training in qualitative research, maintained ongoing reflexivity through documentation in a memo trail and field notes. A relationship was established with participants prior to the study commencement. The participants were informed about the researchers’ personal goals, reasons for conducting the research. All interviews were audio-recorded. Each participant received $75 CAD for the interview. Inview guide was pilot tested. Key interview questions were: What do you know about the prevention of HIV or sexually transmitted infections (STI)? How would you suggest focusing on sexual health within the local community? Can you tell me about your experiences visiting any healthcare service for sexual health concerns?

### Data analysis

All interviews were transcribed verbatim. All transcripts were imported into qualitative coding software, MAXQDA [37]. Our analytical procedure involved careful line-by-line reading and analysis of the transcripts, and reflexive thematic analysis [38]. Thematic analysis involved delineating units of meaning from the data, clustering units of meaning to form thematic statements, and extracting themes. The research team carefully read through all the transcripts, line-by-line, to identify and annotate initial codes based on their interpretation of the text. Next, the team undertook an iterative and inductive process to identify patterns and connections between codes. This involved categorizing codes into categories, and then mapping these categories to overarching themes. Finally, the research team refined and named the identified themes. Coding and analysis was done individually by all members of the research team and checked for consistency in a number of research team meeting discussions. Three member checking meetings occurred with CAC and service providers to inform the themes emerging from the interviews, and to explore and improve the credibility of study data. Social-ecological framework [4–18] was used for the purposes of sifting, sorting, and interpreting data.

Social-ecological framework was employed as a guiding tool for sifting, sorting, and interpreting the data. The framework served as a scaffold, allowing us to weave together the threads of individual, interpersonal, institutional, and structural influences on the sexual health of migrant communities. Reflexivity [39] was maintained through on-going documentation in memo trail and field notes.

## Results

### Study participants

Among a total of 34 individuals: 10 individuals (29%) were African, 6 (18%) Middle Eastern, 5 (15%) Caribbean, 4 (12%) East Asian, 3 (9%) Southeast Asian, 2 (6%) South Asian, and 2 were Latin American. By gender, there were twenty-three women, ten men, and one non-binary person. The age range of participants was 22–54 years old (average age was 35). Among all respondents, 10 were Canadian citizens, 10 were permanent residents, 6 were refugees, 4 were in Manitoba on work permits, and 4 were international students. The median number of years individuals lived in Canada was 4 years.

### Overview of findings

The study revealed the individual, interpersonal, institutional and socio-structural factors that played a role in the sexual health of study respondents. The study found that at the individual level, HIV/STBBI knowledge was influenced by various factors, such as country of origin, education level, length of time in Canada, and involvement in community-based services. Some participants expressed uncertainty about where to access HIV testing in Canada. Risks at the interpersonal level were shaped by contextual factors within one’s microsystem, and accessing sexual health information was identified as an issue within the exosystem of services available to migrants. The interplay between healthcare and language barriers, emerged as significant socioecological factors that shape the sexual health of migrants at the institutional level. Cultural, gender, religious, and social norms played a significant role in discouraging communication about sex, safer sex practices, and birth control. HIV stigma was identified as a recurring theme, underscoring macrosystemic influence on migrants’s sexual health. Participants expressed a need for sexual health information to be provided in English and other languages spoken by migrants in health clinics and community agencies serving migrants, as well as targeted sexual health awareness campaigns for newcomers. The study highlights the complex interplay of factors that influence the sexual health of migrants.

Healthcare Connections Shape Individuals’ HIV/STBBI Awareness and Testing Practices.

Within this theme, participants’ responses highlight the pivotal role of healthcare connections in shaping individuals’ HIV/STBBI awareness and testing practices. At the individual level, differences in HIV/STBBI knowledge appear to be influenced by various factors, including country of origin (with migrants from HIV-endemic countries generally exhibiting greater knowledge), education level, length of time in Canada, and involvement in community-based services in Manitoba. Some respondents, particularly new immigrants, expressed uncertainty about where to access HIV testing in Canada, as one 24-year-old, female participant stated, “I don’t really know where to go…for sexual health services” (Participant 35). On the other hand, those who had a regular doctor or family physician demonstrated good knowledge about HIV and STBBI, and reported undergoing regular testing. For instance, individuals connected to regular doctors discussed the use of pre-exposure prophylaxis (PrEP), a prescription used to prevent HIV, as one 22-year-old African man shared, “PrEP, I found out through my doctor, and he was mentioning to me, and he started telling me about the benefits of PrEP. He gave me some really good options, and I thought about it few months, and then I decided to take it” (Participant 21). Similarly, those with a family physician described the straightforward process they would follow for getting tested, as one 34-year-old, female participant explained, “I will just take my requisition form from my family physician, and I will go to the lab and they take my blood, and I am done” (Participant 01).

### Risks embedded in interpersonal sexual practices

Within this theme, the findings reveal that risks at the interpersonal level were nuanced, shaped by a spectrum of risk scenarios and contextual factors within one’s microsystem, which encompasses the type of partnerships individuals had. As one Caribbean male participant, aged 42, shared, “Right now, I have a second partner. It’s my wife and her…I don’t have anyone else. My wife knows her as well. She doesn’t have other partners. But still we don’t know the person’s previous stories and past sexual events” ( Participant 16). Similarly, other participants drew connections between their sexual risks and the nature of their relationships. For instance, a 31-year-old South Asian male participant reflected, “Me and my wife, we have an open relationship. Depending on how we connect with other people…we have to always be asking ourselves…Okay, that time it was a mistake, you should have been more careful, so now it’s better to get tested” (Participant 20). Interestingly, some participants also relied on their knowledge of advances in HIV biomedical prevention to inform their sexual practices with partners who were living with HIV. As a 29-year-old Caribbean woman shared, “My partner, he is HIV positive…but he takes his meds every day, and he’s undetectable. And that trust of him going out of his comfort zone and telling me that he is positive but undetectable, I felt safer. I would rather sleep and have unprotected sex with a man who will tell me that they are positive but undetectable, instead of going to sleep with somebody who tells me that they are clean” (Participant 13).

### Navigating sexual health information needs, language, and access challenges

A recurring theme among participants was the challenge of accessing sexual health information, which was identified as an issue within the external environment of services available to migrants. Many expressed the need for sexual health information to be provided in English and other languages spoken by migrants in health clinics and community agencies serving migrants. As one participant, a 25-year-old man from Southeast Asia, stated eloquently, “I think having sexual health services in immigrant centers and all doctors’ offices, and just having the information available in many places and languages would really benefit immigrants” (Participant 31).

Participants also highlighted the lack of targeted sexual health awareness campaigns for newcomers, with one 40-year-old African woman stating, “I have attended a lot of campaigns related to children’s issues among newcomers in Canada. But I have never seen any sexual campaign or awareness campaigns for newcomers” (Participant 29). This gap in sexual health information was also noted in social service organizations, with a 23-year-old man from the Caribbean stating, “I don’t remember [agency name] having anything about sexual health. Agencies like that can do more in that regard. Even if they don’t provide that information, they can partner with other agencies that do, or just pass you along to the agencies” (Participant 14). Another important aspect that emerged from the study was the issue of heteronormativity and cisnormativity in sexual health education targeted for migrant communities. As one 29-year-old South Asian gay man expressed, “Whenever immigrants have sex education, it’s so focused on straight people, right, and straight people way of sex” (Participant 17). This highlights the need for inclusive and culturally sensitive sexual health education that addresses the diverse sexual orientations and gender identities within migrant populations.

At the institutional level, the interplay between healthcare and barriers to care has emerged as significant socioecological factors that shape the sexual health of migrants. Language, in particular, has been identified as a crucial element in these barriers to care. One 31-year-old, Asian male participant expressed this challenge eloquently, saying, “Actually… my language… it’s good. But sometimes it’s not good. I might just have a difficulty with vocabulary… So, sometimes the doctors couldn’t understand my questions. I couldn’t explain my question just exactly how I want. They have to improve the translator service” (Participant 13). Similarly, another 41-year old, Middle Eastern, male participant highlighted the impact of language on accessing care, stating, “At the beginning we had a family doctor that only spoke English, so we had to get an interpreter. But after because of the language barrier… we got a new family doctor that was an Arabic speaker” (Participant 18). Another 33-year old Latina respondent emphasized the broader challenges stemming from language and cultural differences, stating, “The language barrier was my biggest concern when I got here… also people don’t have the same reference like you. The culture is different. So, it’s not only about the language barrier” (Participant 10).

Some participants also shared experiences of differential treatment in healthcare based on their ethnicity or migration status, with reports of anti-Black racism as well as immigrants being treated differently in hospitals. This disparity in treatment was seen as a barrier to accessing appropriate care, with one 37-year-old, African female participant stating, “I see Black people and immigrants being treated differently in the hospital. They’ve told me their stories of how healthcare providers don’t give them time to express themselves. That is why sometimes we now go with interpreters to make sure that interpreters advocate for their clients. But I’ve seen clients who complain that the services that were given to them are different from the services that were given to their white counterparts” (Participant 19).

Furthermore, experiences of racial discrimination within institutional contexts were found to have a significant impact on migrants’ engagement in healthcare. Participants shared instances where they felt discriminated against due to their accent or country of origin, leading to feelings of mistrust and discomfort. As one participant, a 31-year-old African woman, shared, " I had to get a yearly check-up, and the woman asked me where I was from. I have lived here for a long time in Canada, but because of my accent, she assumed maybe I had just arrived…Right away, because I’m from [country], she assumed that I have to get an HIV test… I was like, I’ve lived here for a long time, and I’m very safe. In all my relationships, I’ve been very careful, so I got offended a little bit… right away, she was like, well, we have to give you a HIV test… It’s good to be safe. It’s good to be tested. I’m not against testing and all of that kind of stuff. It’s just the way it happened, it hurt my trust” (Participant 24).

### Gender, social norms, and HIV stigma impact sexual health

Within this theme, the exploration of socio-structural factors reveals how cultural, gender, and religious norms shape migrants’ sexual health experiences, impacting communication, attitudes toward sex, and the pervasive influence of HIV stigma within ethnocultural communities and the broader Canadian context. At socio-structural level, migrants’ sexual health was intricately linked to the social macrosystem, encompassing gendered, cultural, and religious social norms. Cultural, gender, and religious norms played a significant role in discouraging communication about sex, safer sex practices, and birth control. As a 24-year-old Southeast Asian woman revealed, “I grew up in [country] and went to a Catholic school. Premarital sex is not allowed, and they’re really against any forms of birth control. That’s why when you start doing it [sex], you have no idea how to stop infection from being passed or how to prevent getting pregnant” (Participant 34).

Furthermore, cultural and gender norms can contribute to sexual stigma and sex-negative attitudes, as exemplified by a quote from a 31-year-old woman from a Latin American country. She expressed how people, especially females, may refrain from discussing their sexual activity or seeking information due to fear of judgment or stigmatization from their community: “I see people being sexually active and not talking about it because they care too much about the way they will be seen by others or by the community… It is stigma, especially for females. I feel like they are more judged in the community and stuff. They will just keep things to themselves, even if they are sexually active, even if they have questions or concerns” (Participant 15).

The experience of HIV stigma also emerges as a recurring theme, underscoring how macrosystems influence migrants. Some migrants living with HIV strategically choose not to disclose their status within their ethnocultural community but may feel more comfortable sharing it in the broader or mainstream Canadian context, as they hope for acceptance, care, and a lack of discrimination. As 41-year-old, African woman expressed, “What would make me open up and share my HIV status is that I’m going to be accepted, I would not be discriminated against. I will still be loved and cared for; I won’t be castigated or judged… In the Canadian context, I think I would be able to share with some people because I know how they feel about it. But maybe not with my community for now” (Participant 32).

The issue of HIV stigma is further underscored by a 52-year-old African woman, who highlighted the rarity of individuals openly discussing their HIV status. She remarked, “I met a lady who just walked in and said, ‘I need my medication because I’m HIV-positive and I have to take my medication at 12:00.‘ She was open to tell me this. How many people are that honest with you, to tell you about HIV? Again, I don’t think the stigma is much here. Back home, the stigma is still very high. So, if the stigma is also not high here, then people are able to talk. We have work to do in terms of getting people to that level where they could tell you, ‘I am HIV-positive, this is my status’” (Participant 22).

## Discussion

Our research constituted an exploration into the socio-ecological landscape of sexual health among migrants residing in the province of Manitoba in Canada. Through a comprehensive investigation, we were able to unveil an array of factors that operate across various levels, ranging from individual-level determinants to the intricate workings of the microsystem encompassing interpersonal dynamics, and the exosystem related to the institutional facets of service provision. Our study also shed light on the crucial role of the macrosystem in shaping the sexual health outcomes of migrants, particularly in the form of negative social norms, oppressive forces, and systemic barriers that hinder their wellbeing.

At the individual level, discernible disparities in first-hand knowledge pertaining to sexually transmitted and blood-borne infections, as well as HIV, were detected among certain newly-arrived immigrants who remained uninformed about the locations offering HIV/STBBI testing in Canada. Such findings resonate with previous research conducted in the United States [38], demonstrating that differences in HIV/STBBI awareness and knowledge are influenced by a host of individual-level variables such as country of origin, level of education, duration of residency in the host country, and level of engagement with healthcare services. Our Canadian study serves to further expand upon this existing line of research. These findings point out the importance to continue increasing awareness, improving access to HIV/STBBI care cascade (including testing), and linking these communities to services. Initiatives to increase HIV/STBBI testing can focus on ensuring adequate safeguards for refugees and people without legal status in Canada [39], and providing culturally safe and linguistically congruent services. Currently, in Canada, immigrants may face a 3-month waiting period for provincial coverage, while refugees access provincial healthcare and the Interim Federal Health Program [40]. International students are covered by the Manitoba International Student Health Plan.

The findings on the interpersonal level of sexual practices shed light on the complex nature of sexual risks among migrant communities in Manitoba. It highlights the importance of considering the nuances of sexual practices within the context of specific relationships, including the type of partnership and communication between partners. The fact that some participants were able to rely on their knowledge of advances in HIV biomedical prevention to inform their sexual practices with partners who were living with HIV shows that education and awareness campaigns can play a crucial role in reducing sexual health risks among migrants. From that perspective, this study is constent with other research [41] showing the importance of education and awareness campaigns in helping increase knowledge and understanding of HIV prevention, care, and treatment strategies, as well as promoting safer sexual practices, and reducing HIV risks among migrant populations.

At the institutional level, this study identified that there was a need for linguistically congruent sexual health information for new immigrants at health agencies, doctors’ offices, and community-based service agencies. These findings are consistent with Canadian research [42] that highlighted the lack of comprehensive sexual health education and language barriers among South Asian immigrants. These findings also highlight the need to build alliances and create platforms for inter-sectoral and interprofessional collaboration between the settlement and health sectors provincially.

At the socio-structural level, this study identified cultural, religious, and gendered norms which exerted significant influence on migrants’ sexual health, including condom use, communication between sexual partners, safer sex practices, and access to birth control. Our research is congruent with previous studies [43, 44] that documented how heterosexual women must navigate gendered norms in sexual negotiations to convince their sexual partners to use condoms.

At institutional and socio-structural level, our study findings also showed the negative impact of sexual stigma, HIV stigma, and racism. Our findings are consistent with Canadian research [45] already showed that racialized migrants living with HIV face stigma and discrimination in both mainstream Canadian society as well as their own ethno-racial communities. Our findings are also consistent with other literature on stigma and HIV disclosure, that speaks how individuals strategically disclose HIV status within their communities [46]. Given the connection between sexual stigma and gender norms, community-based approaches and public awareness campaigns aimed at sexual de-stigmatization can identify strategies for transforming inequitable gender norms to improve uptake of sexual health-related prevention.

Our findings corroborate with previous research [47, 48] which showed that lack of migrant-friendly health settings, racial discrimination, and stigma affect the engagement of migrant communities in healthcare. The perception of HIV stigma and racial discrimination in healthcare contexts negatively affected engagement with sexual health services. Service providers need to develop strategies to eliminate the negative impacts of discrimination these communities experience in services settings. Migrant communities need access to safe, non-judgmental, culturally responsive healthcare and social programs from providers who understand their needs. Policy makers and funders therefore should continue support funding for programs specifically designed for migrant communities in Canada.

Based on the findings from the study, it is also possible to hypothesize about the potential interactions between the individual, interpersonal, institutional, and socio-structural factors that affect the sexual health of migrants. For example, individual-level knowledge and awareness can in turn impact sexual practices and decisions about risk-taking at interpersonal level, where individuals who lack knowledge about sexual health may be more likely to engage in risky sexual behaviors, which can lead to increased rates of HIV or STBBI infection. Similarly, language barriers can make it difficult for individuals to access sexual health information and services, which can contribute to gaps in knowledge and potentially lead to increased risk of HIV or STBBI infection. Interactions between these socio-ecological factors can be complex and vary based on social, economic, and polical circumstances. Overall, these findings suggest that addressing the sexual health needs of migrants requires a multifaceted approach that considers individual, interpersonal, institutional, and socio-structural factors. Public health policies and interventions that address these socio-ecological factors holistically can help to improve sexual health outcomes for migrants and reduce health disparities.

### Limitations and directions for future research

Due to the exploratory nature of this qualitative study and the heterogeneity of respondents, it would be inappropriate to generalize about any group or make cultural comparisons. Future research should also consider other socio-ecological factors that were not examined in this study. For instance, in addition to sexual health, a range of socio-ecological factors at the individual, institutional, and structural levels significantly influenced the health and well-being of migrants in Manitoba. Many migrants in Manitoba encountered challenges in finding safe and affordable housing, citing issues related to credit history as a major obstacle. Respondents, particularly international students, expressed concern about economic challenges and the need to tightly budget for food and other essentials while dealing with the high cost of rent and various financial obligations. However, this study did not examine how economic and housing challenges can impact migrants’ sexual health, such as through limited access to care, increased stress and mental health issues, engaging in high-risk sexual practices, or limited access to safe housing. Future research should investigate the relationship between economic and housing factors and sexual health among migrants in Canada. The current study also considers immigrants and refugees as a single group (i.e., migrants/foreign-born/non-Indigenous). This aggregation weakened the ability to detect important heterogeneity within a diverse population. There is now growing evidence that the health of immigrants and refugees is influenced by when, where, and how they migrated. Future research on this topic also needs to consider the heterogeneity of these populations by designing studies that attend to the health needs of specific migrant groups. Another limitation of this study is its exclusive focus on the socio-ecological framework. This research did not use other conceptual and methodological frameworks, such as transnationalism, intersectionality, and social determinants of health, to explore the intersection between migration and sexual health inequities among migrants in this study.

## Conclusions

To the best of our knowledge, this is the first socio-ecological study examining sexual health among migrant communities in Manitoba, Canada. Our findings contribute to the limited literature on the sexual health of migrants in an urban Canadian setting by demonstrating the multiple socio-ecological factors that influence sexual health among migrants in Manitoba. Public health interventions aimed at improving sexual health outcomes for these communities must consider all these systems and be targeted at multiple levels. Overall, the study highlights the need for a multi-level, integrated approach to address the individual, interpersonal, institutional, and socio-structural factors that affect the sexual health of migrants. This approach should involve collaboration between public health and health care providers, social service organizations, and community-based agencies to develop culturally sensitive and inclusive sexual health education and awareness campaigns, provide access to language-appropriate sexual health services, and facilitate the involvement of migrants in community-based services. Addressing the diverse set of socio-ecological elements identified in this study can reduce inequities in access to sexual health information and culturally affirming services for migrants.

## Data Availability

The datasets used and analysed during the current study available from the corresponding author on reasonable request.
